# Sulforaphene Attenuates *Cutibacterium acnes*-Induced Inflammation

**DOI:** 10.4014/jmb.2209.09051

**Published:** 2022-10-30

**Authors:** Hwan Ju Hwang, Jong-Eun Kim, Ki Won Lee

**Affiliations:** 1 WCU Biomodulation Major and Research Institute of Agriculture and Life Sciences, Department of Agricultural Biotechnology, Seoul National University, Seoul 08826, Republic of Korea; 2 Department of Food Science and Technology, Korea National University of Transportation, Jeungpyeong 27909, Republic of Korea

**Keywords:** Acne, *Cutibacterium acnes*, inflammation

## Abstract

Acne is a chronic inflammatory disease of the sebaceous gland attached to the hair follicles. *Cutibacterium acnes* is a major cause of inflammation caused by acne. It is well known that *C. acnes* secretes a lipolytic enzyme to break down lipids in sebum, and free fatty acids produced at this time accelerate the inflammatory reaction. There are several drugs used to treat acne; however, each one has various side effects. According to previous studies, sulforaphene (SFEN) has several functions associated with lipid metabolism, brain function, and antibacterial and anti-inflammatory activities. In this study, we examined the effects of SFEN on bacterial growth and inflammatory cytokine production induced by *C. acnes*. The results revealed that SFEN reduced the growth of *C. acnes* and inhibited proinflammatory cytokines in *C. acnes*-treated HaCaT keratinocytes through inhibiting NF-κB-related pathways. In addition, SFEN regulated the expression level of IL-1α, a representative pro-inflammatory cytokine expressed in co-cultured HaCaT keratinocytes and THP-1 monocytes induced by *C. acnes*. In conclusion, SFEN showed antibacterial activity against *C. acnes* and controlled the inflammatory response on keratinocytes and monocytes. This finding means that SFEN has potential as both a cosmetic material for acne prevention and a pharmaceutical material for acne treatment.

## Introduction

**Fig. 1:**
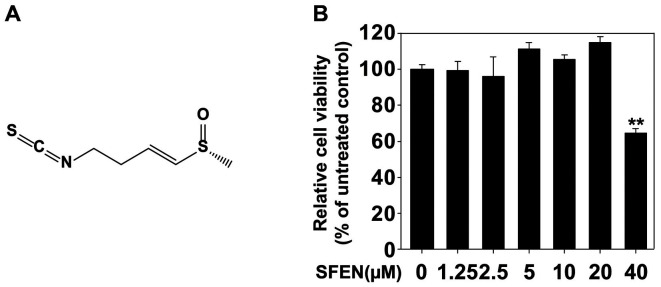
Effects of sulforaphene (SFEN) on HaCaT cell viability. (**A**) Chemical structure of SFEN. (**B**) MTT assay results showed that SFEN did not exhibit cytotoxicity until 20 μM concentration. All graphs represent the means ± SEM (*n* = 3). Asterisks indicate a significant inhibition by SFEN compared with non-treated group (**p < 0.01) using Student’s *t*-test.

Radish seeds have long been used in East Asian traditional medicine for antibacterial purposes and to treat intestinal and skin inflammation [1]. Sulforaphene (SFEN, Fig. 1A), an active compound in radish seeds and a highly studied phytochemical, is a material that gives sulforaphane its carbon double bonds [2-4]. Additionally, SFEN is involved in lipid metabolism [5], has antibacterial effects on different types of bacteria [6], and also alleviates inflammatory diseases, including cancer [5, 7-10].

Acne is a chronic inflammatory disease of the sebaceous glands, usually starting in puberty and disappearing in a person’s mid-20s [11]. Clinically, it appears in the form of comedones, pustules, cysts, and nodules [12]. It mainly appears on the face, neck, and chest where there is a lot of sebum secretion. As a result, it sometimes leaves unpleasant scarring on the skin [13]. The condition is not only a cosmetic concern, it also causes various psychological problems [14]. Acne occurs for a variety of reasons. In women, acne sometimes worsens periodically [13], usually about 1-2 weeks before menstruation, and it is thought to be caused by a progesterone hormone. Patients with endocrine disorders are particularly prone to acne [15], while various additives in cosmetics can also be a cause [16]. Physical and mental stress, such as lack of sleep and overwork, can exacerbate acne by increasing the secretion of androgens [17]. Acne worsens in strong sunlight or in hot, humid environments. If an affected individual constantly touches, rubs, or presses on acne, secondary infection and spread of the condition can also occur [13].

During puberty, an excess of male hormone can activate secretion of the sebaceous glands, and the epithelium of the hair follicles is complete and immature keratinization occurs, which is an abnormal keratinization called heterokeratosis [18]. Among the bacteria resident in hair follicles is *Cutibacterium acnes*, which secretes a lipolytic enzyme that decomposes triglycerides in sebum to form free fatty acids and stimulate hair follicles. In addition, the immunological response to *C. acnes* contributes to the inflammatory response of acne [17]. Acne that has been severely stagnant is unsightly, but it is highly likely to remain as a scar, so it is treated with injection therapy to reduce the occurrence of scars [19]. When acne is spread widely, chemical peeling is used to exfoliate dead skin cells and temporarily open clogged pores, reduce inflammation, and increase skin absorption of other medications, thereby enhancing the efficacy of the drug. While physical treatment is expensive and painful [12], current drug therapy against acne aims to suppress *C. acnes* proliferation, control sebum secretion, and prevent hyperkeratinization and inflammatory reaction. Topical treatments include antibiotics, retinoids, benzoyl peroxide, and azelaic acid [20]. These are often accompanied by side effects such as dryness, contact allergic reaction, erythema, epidermal deprivation, burning sensation, and skin irritation [12]. Systemic therapy includes antibiotics, isotretinoin, and hormone preparations, which can also cause diarrhea, nausea, dry skin, depression, vomiting, and headaches [21]. As such, existing acne treatments are limited due their various side effects [22]. Therefore, in recent years, attempts have been made to develop acne treatments that lessen these side effects. In this study, we reveal the efficacy of treating acne by SFEN using HaCaT cells, which are keratinocytes of the skin. The mechanism of action was also elucidated.

## Materials and Methods

### Chemicals and Reagents

SFEN was obtained from LKT Laboratories, Inc. (USA). Among the antibodies, p-IκBα (Ser32) and p-IKKα/β (Ser176/180) were obtained from Cell Signaling Biotechnology (USA). Other antibodies were obtained from Santa Cruz Biotechnology, Inc. (USA). DMEM, RPMI-1640 and fetal bovine serum (FBS) were obtained from Welgene, Inc. (Korea). Brain heart infusion broth, a GasPak system, and brain heart infusion agar (BD Biosciences, Inc., USA) were used to culture *C. acnes*. A protein quantification kit was purchased from Bio-Rad. Other chemicals were purchased from MilliporeSigma, Inc. (USA).

### Cell Culture

HaCaT cells were cultured in DMEM with 10% FBS at 5% CO_2_ and 37°C. RPMI-1640 medium is used for culture of Human THP-1 promonocytic cells. Each cell was seeded and, when it reached 80% confluence, was replaced with serum-free media for starvation. After 1 h of treatment with SFEN, heat-killed *C. acnes* (multiplicity of infection [MOI] = 100) was added to the medium. In co-culture experiments, 1.0 × 10^5^ cells/well of HaCaT cells were seeded in the upper chamber of a 12-well Transwell (Corning Inc., USA). THP-1 cells were seeded in the lower chamber at 2.0 × 10^5^ cells/well. Then, following treatment with SFEN (5, 10, and 20 mM), heat-killed *C. acnes* (MOI = 100) was treated 1 h later.

### Bacterial Culture

Brain heart infusion broth is used for culturing *C. acnes* *ATCC 6919* under anaerobic conditions. A GasPak system was used to produce anaerobic conditions. The pellets were treated at 65°C for 30 min before being dissolved in DMEM.

### Luciferase Reporter Gene Assay and Enzyme-Linked Immunosorbent Assay (ELISA)

pGF-NF-κB-mCMV-EF1-Puro was purchased from System Biosciences, Inc. (USA). A Luciferase Assay Kit was also obtained (Promega Inc., USA), as were human IL-1β, human *IL-6*, and a Human *IL-8*/CXCL8 DuoSet ELISA Kit (R&D Systems, Inc., USA). All items were used according to the manufacturer’s instructions.

### qPCR

RNAs from HaCaT cells were prepared by using RNAiso Plus (Takara Bio Inc., Japan). The concentration and purity of the RNAs were measured using a NanoDrop ND-2000 spectrophotometer (Thermo Fisher Scientific, USA). A PrimeScriptTM 1st Strand cDNA Synthesis Kit (Takara Bio Inc.) was used for reverse transcription. IQ SYBR (Bio-Rad Laboratories Inc., USA) was used for RT-PCR. cDNA (2 μl) was used in triplicate with *GAPDH* as an internal control. cDNA was amplified using the following primers: *IL-8* forward (5’- TCT TGG CAG CCT TCC TGA TT -3’), *IL-8* reverse (5’- TTT CGT GTT GGC GCA GTG T -3’); *IL-6* forward (5’- CAA TCT GGA TTC AAT GAG GAG AG -3’); *IL-6* reverse (5’- CTC TGG CTT GTT CCT CAC TAC TC -3’); *GAPDH* forward (5’-TCC TCA CCC TGA AGT ACC CCA T –3’); *GAPDH* reverse (5’- AGC CAC ACG CAG CTC ATT GTA -3’).

### Western Blot Assay

Protein lysates (60 mg) were separated by SDS-PAGE and transferred onto a PVDF membrane (MilliporeSigma, Inc.). The membrane was blocked in skim milk over 2 h and then incubated with an indicated primary antibody over 6 h. After washing 3 times, hybridization was carried out with an HRP-conjugated secondary antibody. A chemiluminescence detection kit from MilliporeSigma, Inc. was used for protein bands.

### Statistical Analysis

One-way analysis of variance (ANOVA) and post-hoc Tukey's test were used. *p*-values < 0.05 were considered statistically significant. IBM SPSS Statistics v.23.0 (IBM, UAS) was used for statistical analysis. The data were expressed as means ± standard error of the mean (SEM).

## Results

### Effects of SFEN on *C. acnes* Growth

An MIC test was performed to determine the antibacterial effect of SFEN. The strains used for MIC were *C. acnes*, *E. coli* CCARM 9008, CCARM 3102, or *S. aureus* CCARM 3102 (Table 1). In particular, SFEN inhibited the growth of *C. acnes* more so than that of other bacteria. Therefore, SFEN exhibits a direct antibacterial effect against *C. acnes*.

### Effects of SFEN on Proinflammatory Cytokines in *C. acnes*-Treated HaCaT Keratinocytes

**Fig. 2:**
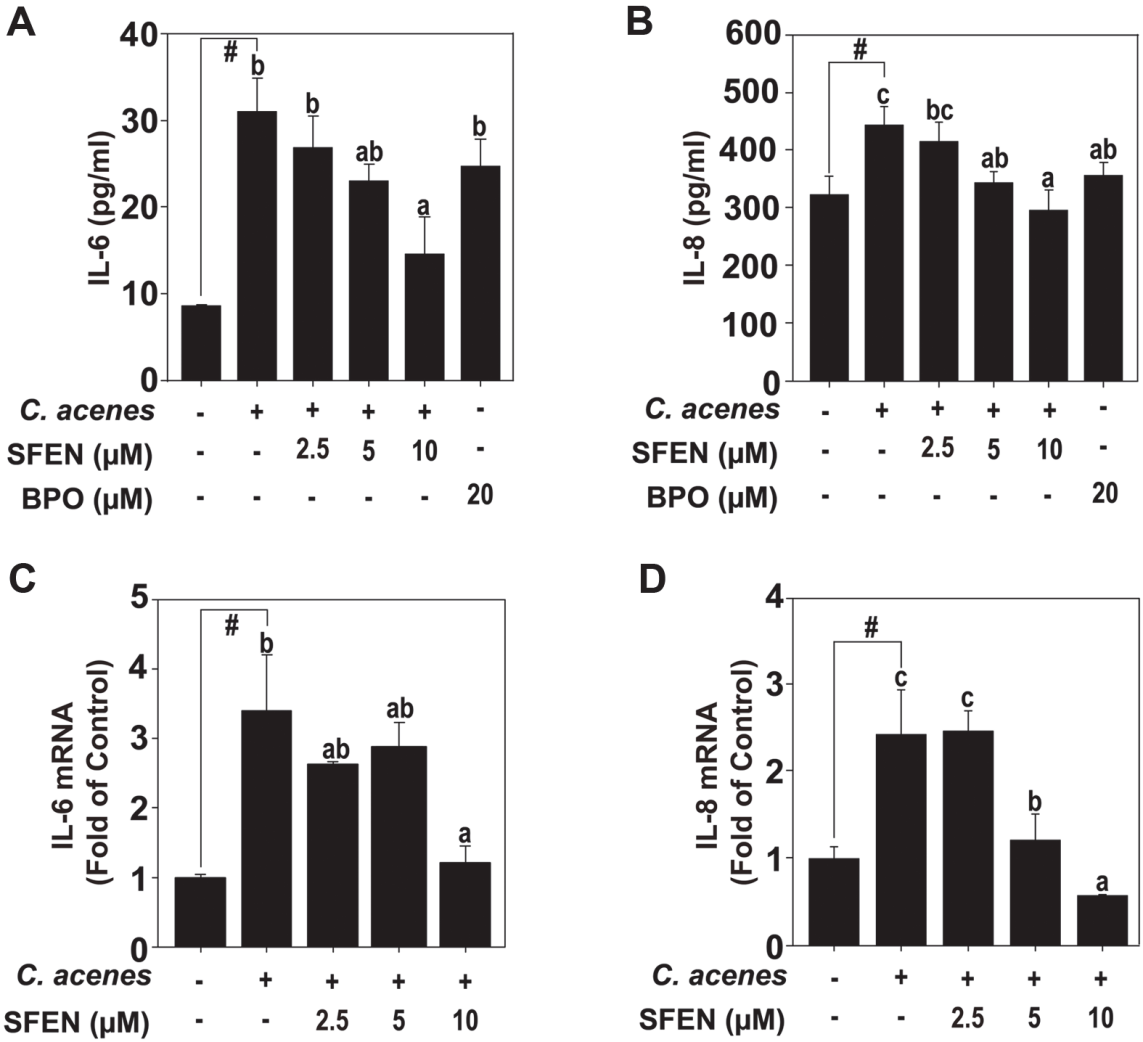
Effects of SFEN on the production of pro-inflammatory cytokines in HaCaT cells. The secretion levels of (**A**) *IL-6*, and (**B**) *IL-8* were detected by ELISA. The expression levels of (**C**) *IL-6* mRNA, and (**D**) *IL-8* mRNA were detected using qPCR described in Materials and Methods. Bars marked with different letters (a–c) are significantly different (*p* < 0.05) according to Tukey's test. The hashes (#) indicate a significant difference (*p* < 0.05) compared to untreated control.

To conduct the experiment at a concentration without cytotoxicity, the MTT assay was performed. As in Fig. 1B, SFEN shows decreased viability at concentrations above 40 μM. Therefore, another experiment was conducted with an SFEN concentration of less than 20 μM, which does not affect cell death. The protein levels of *IL-6* and *IL-8*, the major cytokines produced by *C. acnes*, were measured through ELISA. SFEN effectively inhibited these cytokines (Figs. 2A and 2B). As in Figs. 2C and 2D, the mRNA levels of *IL-6* and *IL-8* were also effectively inhibited by SFEN.

### Effects of SFEN on NF-κB Signaling Pathway Inhibition in HaCaT Keratinocytes

**Fig. 3:**
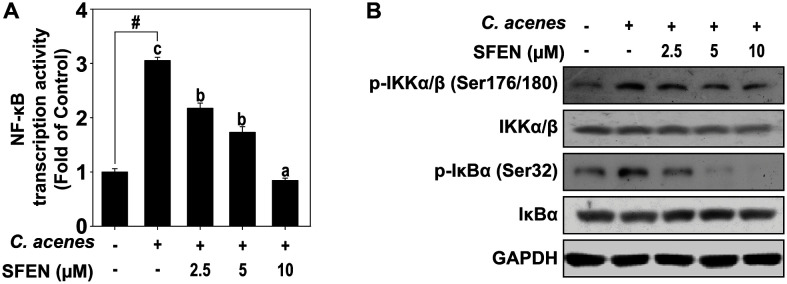
Inhibitory effects of SFEN on the transcription activity of NF-κB and the upstream regulator proteins of NF-κB. (**A**)Transcription activity of NF-κB was measured by luciferase reporter gene assay described in Materials and Methods. Bars marked with different letters (a–c) are significantly different (*p* < 0.05) according to Tukey's test. The hashes (#) indicate a significant difference (*p* < 0.05) compared to untreated control. (**B**) Phosphorylated and total forms of IKKα/β (Ser176/180) and IκBα (Ser32) proteins were determined by western blot assay as described in Materials and Methods.

The transcription factor that plays a crucial role in the expression of *IL-6* and *IL-8* is NF-κB in HaCaT keratinocytes [23, 24]. We used luciferase reporter gene assays to investigate the activation of NF-κB transcription factors. In HaCaT cells transduced with NF-κB reporter plasmid, treatment with *C. acnes* increases the activity of luciferase, and treatment with SFEN decreases it (Fig. 3A). In addition, SFEN inhibited the phosphorylation of IKKα/β and IκBα (Fig. 3B), which are upstream regulators of NF-κB.

### Effects of SFEN on *C. acnes*-Induced IL-1β in Cocultured HaCaT Keratinocytes and THP-1 Monocytes

**Fig. 4:**
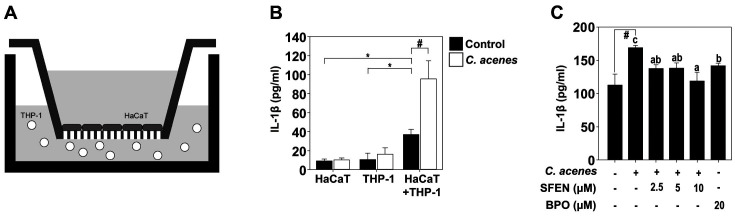
Effects of SFEN on the production of IL-1β cytokine in a coculture model of HaCaT cells and THP-1 cells. (**A**) The coculture method of HaCaT cells and THP-1 cells using Transwells as described in Materials and Methods. (B and C) The concentration of IL-1β in conditioned media was detected by ELISA. White bar indicates *C. acnes*-induced group and black bar indicates the control group. Bars marked with different letters (a–c) are significantly different (*p* < 0.05) according to Tukey's test. The hashes (#) indicate a significant difference (*p* < 0.05) compared to untreated control.

An experimental method of coculturing two cells was used to simulate a phenomenon occurring in the human body. In the human body, keratinocytes cause skin inflammation through interaction with Langerhans cells. The THP-1 cell line was originally known as a monocytic leukemia cell, but has characteristics of dendritic cells [25]. To show the protein level of IL-1β in the coculture model (Fig. 4A), the cells were divided into three conditioned groups: HaCaT only, THP-1 only, and HaCaT and THP-1. Heat-killed *C. acnes* was added to each group. IL-1β was increased significantly when HaCaT and THP-1 cells were cocultured (Fig. 4B). In our coculture model, SFEN inhibited IL-1β induced by heat-killed *C. acnes* in a concentration-dependent manner (Fig. 4C).

## Discussion

The sebaceous glands are abundant on the face, back, and chest areas where acne is common. These glands are connected to hair follicles and produce an oily substance called sebum. [14]. Under normal conditions, sebum rises along the hair follicle wall and is discharged through the skin, but when the hair follicle is blocked, sebum cannot be discharged and gets trapped around the hair follicle, and bacteria that cause inflammation grow, which leads to acne [11]. Among the bacteria resident in hair follicles, *C. acnes*, in particular, secretes lipolytic enzymes to form free fatty acids and stimulate hair follicles. It is also known that an immune response to these bacteria contributes to acne inflammation [15]. Therefore, if *C. acnes* can be prevented from growing on the skin and the inflammation caused by it can be alleviated, the damage to the skin caused by acne can be reduced [14]. In this study, we found that SFEN exerts antibacterial activity against *C. acnes* and relieves related inflammation.

*C. acnes* promotes this inflammation through Toll-like receptor (TLR) activation. Among TLRs, TLR2 plays the most important role in inflammation caused by *C. acnes*. Activation of TLR2 by *C. acnes* induces the MAPK and NF-κB pathways. Activated NF-κB transcription factors promote the expression of proinflammatory cytokines [26, 27]. Activation of TLR2 by *C. acnes* induces proinflammatory cytokines such as *IL-8* and *IL-6* in keratinocytes [28]. These cytokines play roles in regulating inflammatory responses in keratinocytes and monocytes [29, 30]. In this study, SFEN inhibited the transcriptional activity of NF-κB by suppressing IkBα and IKKα/β phosphorylation, which regulates NF-κB activation.

There are many reports that *C. acnes* contributes to the production of cytokines that are pivotal in inflammatory acne via a TLR2 pathway [31]. *C. acnes*-induced cytokine production is associated with TLR2 activation. *C. acnes* induces the production of IL-1β, *IL-8*, TNF-α and IFN-γ in keratinocytes [28]. It has been shown that peritoneal macrophages from knockout of TLR6 and TLR1 in mice produce *IL-6* in response to *C. acnes* infection, but not TLR2 knockout mice [32]. Therefore, *C. acnes* activates TLR2 and TLR2 activates NF-κB. Activated NF-κB induces various inflammatory responses. SFEN can block NF-κB activation, thereby preventing inflammation caused by *C. acnes*.

A coculture model of HaCaT and THP-1 cells was used to evaluate the effects of the material more similar to an in vivo situation. The cytokines produced by *C. acnes*-induced keratinocytes act on immune cells in the skin. These cytokines induced other proinflammatory cytokines, such as IL-1β [33]. IL-1β secretion was strongly increased when *C. acnes* was treated in a coculture model than when HaCaT cells and THP-1 cells were treated with *C. acnes*, respectively. When SFEN was treated in a coculture model, IL-1β secretion induced by *C. acnes* was inhibited. This suggests that SFEN could inhibit inflammation occurring at acne sites.

In conclusion, we identified effects of SFEN on the growth of *C. acnes* and *C. acnes*-induced inflammation. Our findings demonstrate that SFEN has potential both as a cosmetic material for acne prevention, and as a pharmaceutical material for acne treatment.

## Figures and Tables

**Table 1:** Minimum inhibitory concentration (MIC) test for SFEN.

Microbial strains	Minimum inhibitory concentration (MIC)		

	SFEN (mM)	Norfloxacin (mM)	Clindamycin (mM)
*C. acnes* CCARM 9008	0.31	-	0.07
*E. coli* CCARM 0012	1.25	0.38	-
*S. aureus* CCARM 3102	1.25	100.21	-
*E. coli* ATCC 25922	1.25	0.19	-
*S. aureus* ATCC 29213	1.25	3.13	-
*S. pneumoniae* CCARM 0031	1.25	-	-
